# Reply to Forni et al., “Multiple Selected Changes May Modulate the Molecular Interaction between *Laverania* RH5 and Primate Basigin”

**DOI:** 10.1128/mBio.00917-18

**Published:** 2018-05-22

**Authors:** Lindsey J. Plenderleith, Weimin Liu, Oscar A. MacLean, Yingying Li, Dorothy E. Loy, Sesh A. Sundararaman, Frederic Bibollet-Ruche, Gerald H. Learn, Beatrice H. Hahn, Paul M. Sharp

**Affiliations:** a Institute of Evolutionary Biology, University of Edinburgh, Edinburgh, United Kingdom; b Centre for Immunity, Infection and Evolution, University of Edinburgh, Edinburgh, United Kingdom; c Department of Medicine, University of Pennsylvania, Philadelphia, Pennsylvania, USA; d Department of Microbiology, University of Pennsylvania, Philadelphia, Pennsylvania, USA; Columbia University

**Keywords:** *Laverania*, *Plasmodium falciparum*, RH5, basigin, chimpanzee, gorilla

## REPLY

We have previously shown that the human malaria parasite Plasmodium falciparum resulted from the cross-species transmission of a parasite (which we have termed Plasmodium praefalciparum) that infects wild-living gorillas (1). In a recent paper (2), we took issue with claims made by Forni et al. in 2015 (3) regarding the origin of P. falciparum. This paper was entitled “Positive selection underlies the species-specific binding of Plasmodium falciparum RH5 to human basigin,” with the final sentence of their abstract stating “Data herein provide an evolutionary explanation for species-specific binding of the PfRH5-BSG ligand receptor pair and support the hypothesis that positive selection at these genes drove the host shift leading to the emergence of P. falciparum as a human pathogen.” These claims were the focus of our criticism, which is why we did not discuss their application of a “phylogenetics-population genetics method to search for sites that were positively selected” in BSG (basigin) genes: from that analysis, the authors reported that “no positively selected sites were found in the human lineage” (3), and so these analyses provided no evidence relevant to their claims.

The data analyzed by Forni and colleagues (3) were insufficient to support the claim that positive selection at BSG and RH5 contributed to the emergence of the human parasite. For BSG, they presented (Table 1 in reference 3) evidence for two positively selected sites. However, the signal of selection was due to the inclusion of many monkey and prosimian sequences in their analysis. These species are irrelevant to our understanding of the recent evolution of P. falciparum, because *Laverania* (the subgenus comprised of P. falciparum and seven other described species [1, 4]) has only been found infecting African apes in the wild. One of the sites (codon 102) identified by Forni and colleagues is conserved among all apes analyzed and so is not pertinent to their claims. The other (codon 27) is conserved in humans, chimpanzees, and bonobos but differs in gorillas (2); again, it is hard to see how this site is relevant to the emergence of P. falciparum following a host jump from gorillas to humans.

**FIG 1:**
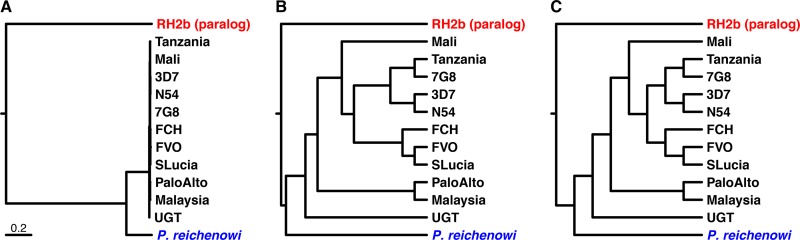
Relationships among the RH5 sequences used by Forni et al. (3), adapted from reference 2. (A) Relationships among the RH5 sequences with branch lengths drawn to scale; the bar denotes 0.2 amino acid replacements per site (2). (B) Relationships among the RH5 sequences as depicted by Forni et al. (3). (C) Relationships among the RH5 sequences without specified branch lengths; branches have unit lengths. P. falciparum strains are shown in black in panels A to C.

For RH5, Forni and colleagues (3) analyzed 13 sequences. However, 11 of these were near-identical alleles from strains of P. falciparum, one was the ortholog from Plasmodium reichenowi (a chimpanzee parasite), and the last was the sequence of a highly divergent paralog (RH2b) from P. falciparum. The relationship among these sequences is shown in Fig. 1A, which illustrates that Forni et al. were considering, in effect, only two sequences (those of P. falciparum and P. reichenowi), with no power to determine whether any differences between those two sequences arose on the branch leading to one species or the other from their common ancestor; given the relationships among P. falciparum, P. praefalciparum, and P. reichenowi (1), only changes that occurred near the tip of the P. falciparum branch could be relevant to the emergence of the human parasite. Strangely, Forni et al. (3) depicted the relationships among these sequences very differently (Fig. 1B). They now suggest (5) that the branch lengths in their tree “were simply not reported,” although a tree with undefined (unit length) branches would look somewhat different (Fig. 1C). Clearly, the branch lengths in Fig. 1B are extremely distorted compared to those in Fig. 1A. We still find it very difficult to explain why Forni et al. chose to depict the tree as they did, because it could mislead readers into thinking there was more information in the data set than was the case.

Forni et al. (5) now state that a paralog (such as RH2b) can be informative in such analyses. This is only true if the paralog is closely related, and indeed one of the papers they cite (6) specifically makes this point. The RH2b protein sequence is only ~27% identical to the RH5 sequences, and therefore is far too distant to be useful. In fact, alignment of RH2b and RH5 requires the insertion of many gaps. Forni et al. identified two sites in RH5, codons 190 and 447, as being positively selected (Table 2 in reference 3), but in their alignment, both are opposite gaps in RH2b. Obviously, for these two sites, the RH2b sequence must be useless in assessing whether the differences between the two species occurred on the lineage leading to P. falciparum or that leading to P. reichenowi.

The difficulty in determining whether any changes at sites within RH5 had occurred during the recent evolution of P. falciparum is exacerbated by the fact that, unbeknownst to Forni and colleagues, the *Rh5* gene in the ancestor of P. praefalciparum and P. falciparum was the product of a lateral gene transfer from the ancestor of another gorilla parasite, Plasmodium adleri, and so is unusually distantly related to the *Rh5* gene of P. reichenowi (7). Given this unusual event and experimental data implicating the interaction between RH5 and BSG as being important for determining the host specificity of *Laverania* parasites (8), the question of the evolution of RH5 was of obvious interest. Thus, we determined the sequences of partial *Rh5* genes from a range of different ape parasites so that we might address the question properly (2). These analyses showed that the two sites identified by Forni et al. are conserved among the *Rh5* genes of P. falciparum and its three closest relatives. Thus, again, the sites claimed by Forni et al. to be involved in the host shift leading to the emergence of P. falciparum cannot have had any relevance to this process. However, we did identify a number of codons in *Rh5* that may have undergone adaptive evolution during diversification of the *Laverania*, including three encoding contact residues involved in the binding of P. falciparum RH5 to human BSG. Most interestingly, one of these sites (codon 197) appears to have undergone an amino acid replacement subsequent to the origin of P. falciparum from P. praefalciparum (2), and thus may have been involved in the emergence of P. falciparum.

In conclusion, there is nothing in the letter by Forni and colleagues (5) that bears on our critique (2) of their previous paper (3). Our quotations from their paper were not mistaken, and our criticisms were not erroneous. The data that they analyzed did not, and indeed could not, support their claims. For RH5, the strange way in which they depicted the relationships among the sequences may have obfuscated the fact that their analyses lacked any power. The data we subsequently generated did allow an appropriate analysis of the recent evolution of the *Rh5* gene in *Laverania* parasites and produced interesting findings (2). Now Forni et al. (5) have reanalyzed our new data, using similar methods, and have (unsurprisingly) obtained similar results.
